# Impact of Chocolate percutaneous transluminal angioplasty balloon on vessel preparation in drug-coated balloon angioplasty for femoropopliteal lesion

**DOI:** 10.1186/s42155-022-00324-z

**Published:** 2022-09-01

**Authors:** Shigemitsu Shirai, Shinsuke Mori, Kohei Yamaguchi, Masafumi Mizusawa, Toshiki Chishiki, Kenji Makino, Yohsuke Honda, Masakazu Tsutsumi, Mana Hiraishi, Norihiro Kobayashi, Masahiro Yamawaki, Yoshiaki Ito

**Affiliations:** Department of Cardiovascular Medicine, Department of Cardiology, Saiseikai Yokohamashi Tobu Hospital, Yokohama, 230-8765 Japan

**Keywords:** Peripheral artery disease, Endovascular procedures, Femoral artery, Popliteal artery, Drug-coated balloon

## Abstract

**Purpose:**

To compare the impact of Chocolate and conventional balloons on vessel preparation in percutaneous transluminal angioplasty.

**Materials and methods:**

This single-center retrospective study included 111 endovascular therapy consecutive cases of femoropopliteal lesions using drug-coated balloon strategy with a 1:1 pre-dilation balloon diameter between February 2020 and August 2021, divided into the Chocolate percutaneous transluminal angioplasty (*n* = 48) and conventional (*n* = 63) groups. Before the availability of Chocolate balloons in Japan (December 2020), a standard semi-compliant or non-compliant balloon was used for vessel preparation. The primary endpoint was rate of severe dissection after pre-dilatation. Secondary endpoints were angiographic percent diameter stenosis, bailout stent rate, primary patency rate, and freedom from target-lesion-revascularization rate at six months.

**Results:**

There was no significant difference in patient and lesion characteristics. The procedural characteristics comprised balloon length 90 ± 37 and 149 ± 95 mm (*P* = 0.004) and inflation pressure 11 ± 3 and 16 ± 7 atm (*P* < 0.001) in the Chocolate and conventional groups, respectively. Regarding primary endpoint, rates of severe dissection were 4.2% and 25% (*P* = 0.003); regarding secondary endpoints, percent diameter stenosis was 18 ± 15% and 20 ± 17% (*P* = 0.409), and the rate of bailout stenting was 2.1% and 15.9% (*P* = 0.016) in the Chocolate and conventional groups, respectively. The primary patency rates at six months were 89.1% and 85.2% (*P* = 0.670), and freedom from target-lesion-revascularization rate at six months was 100% and 92.8% (*P* = 0.691) in the Chocolate and conventional groups, respectively.

**Conclusion:**

Chocolate percutaneous transluminal angioplasty balloons reduce the rate of severe dissection while maintaining a sufficient dilatation effect during drug-coated balloon vessel preparation.

## Introduction

Recent advances in endovascular treatment (EVT) technology and related devices have made EVT the first-line treatment for peripheral arterial disease with femoropopliteal (FP) lesions (Aboyans et al. 2017). Nitinol stents have led to excellent acute lumen gains in the past two decades; however, their primary patency remains unsatisfactory (Schillinger et al. 2006; Iida et al. 2014). For longer procedure durability, paclitaxel-eluting stents were developed to preclude neointimal proliferation in FP lesions by enabling sustained and controlled release of paclitaxel over the first 12 months after stent implantation (Bisdas et al. 2018). Contrastingly, the use of a drug-coated balloon (DCB) is a suggested alternative, especially for popliteal lesions, small vessel diseases, and young claudicants, to overcome the shortcomings of metallic stents (such as in-stent restenosis and fractures) (Tosaka et al. 2012; Scheinert et al. 2005). The Society for Cardiovascular Angiography & Interventions Consensus Guidelines (Feldman et al. 2018) recommends DCB as the final device for FP lesions. Although there is recent evidence of the efficacy of DCB in long FP lesions (Schmidt et al. 2016; Micari et al. 2016), the rate of bailout stenting is not low because of flow-limiting dissection after angioplasty. Fujihara et al. (Fujihara et al. 2017) reported a dissection rate of 42% in severe, flow-limiting dissections (types C–F) post-balloon angioplasty of the superficial femoral artery (SFA); most required subsequent intervention and bailout stenting. To address the high prevalence of dissections and to ensure metal-free interventions, new angioplasty balloon technologies are required.

The Chocolate percutaneous transluminal angioplasty (PTA) balloon catheter is a balloon developed to minimize overall vessel trauma. This balloon is constrained by a nitinol scaffold that subsegments the balloon when inflated, creating valleys and grooves on the balloon surface that increase its contact surface area. This balloon design helps to disperse the force associated with angioplasty along this increased contact surface area, resulting in a controlled and differential dilatation approach to minimize overall vessel trauma (Mustapha et al. 2018). A study showed short-term procedural results without flow-limiting dissections. This strategy minimizes bailout stent use and provides an alternative to primary stenting with excellent 12-month primary patency rates and low rates of target lesion revascularization (TLR) (Mustapha et al. 2018). However, there have been no studies comparing them with conventional balloons. Therefore, this study investigated the impact of a chocolate PTA balloon on vessel preparation for angioplasty and compared it with that of a conventional balloon.

## Materials and methods

### Patients

This single-center, retrospective, nonrandomized observational study was conducted between April 2020 and August 2021. The main strategy for FP lesions in this study was DCB angioplasty, except in cases of severely calcified lesions, thrombotic lesions, or chronic limb-threatening ischemia (CLTI) with a large wound. The Chocolate PTA balloon has been available in Japan since December 2020. Before their availability, a standard semi-compliant or non-compliant balloon was used for vessel preparation.

**Fig. 1:**
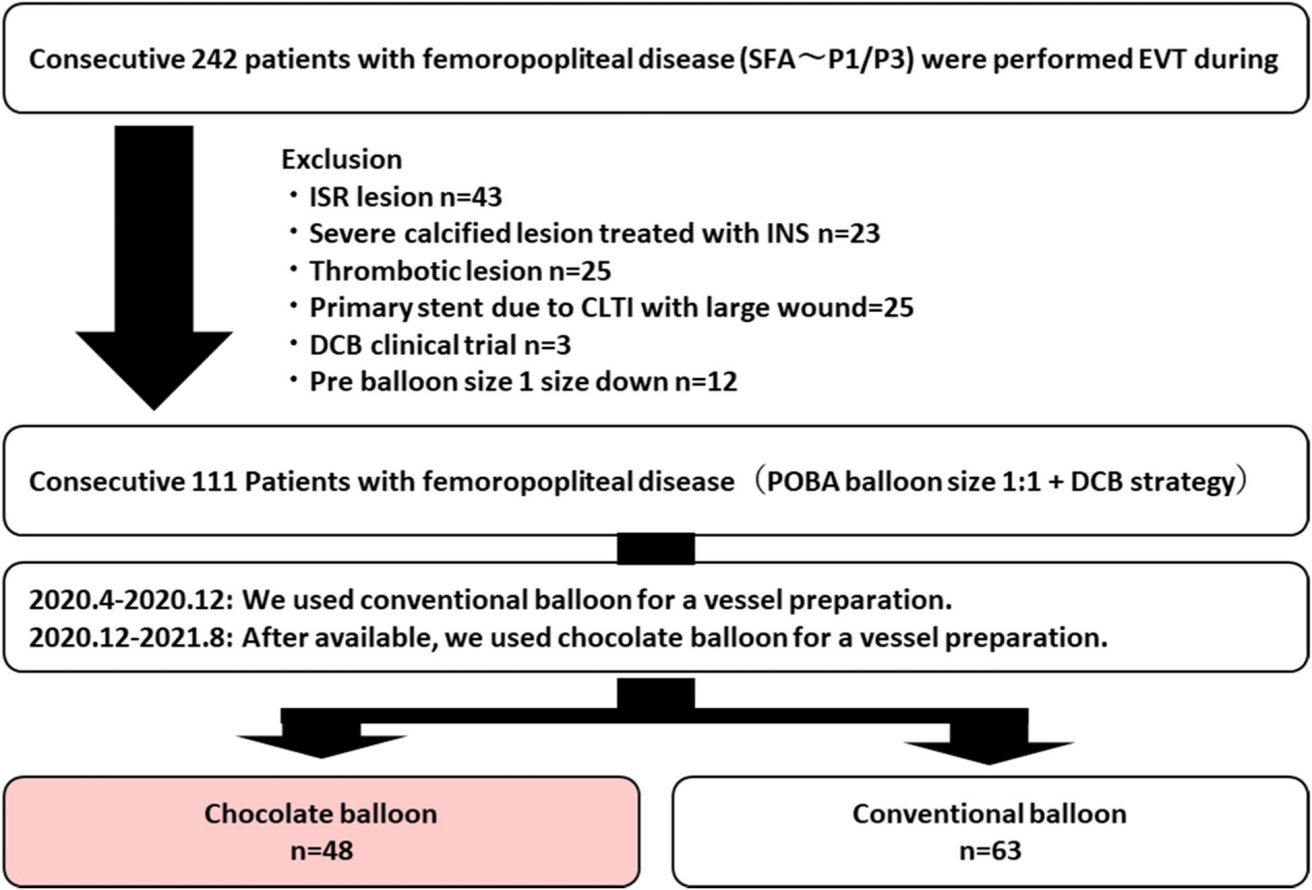
Study flowchart. EVT, endovascular therapy; ISR, in-stent restenosis; INS, interwoven nitinol stent; CLTI, chronic limb-threatening ischemia; DCB, drug-coated balloon; POBA, plain old balloon angioplasty

During the study period, EVT was performed in 242 consecutive cases of FP lesions. The exclusion criteria comprised in-stent restenosis lesions (*n* = 43), clinical trial cases (*n* = 3), thrombotic lesions (*n* = 25), cases with primary stent strategy because of calcified lesions (*n* = 23), and CLTI with large wounds (*n* = 25). To match the diameter of the pre-balloon with the reference vessel size of 1:1, cases (*n* = 12) in which pre-dilatation balloons were selected with a diameter of 1 size down were excluded. According to the type of pre-dilated balloon, 111 patients (111 lesions) were divided into the chocolate PTA (*n* = 48) and conventional (*n* = 63) groups (Fig. 1).

All the patients had symptoms corresponding to categories 2–6 of Rutherford’s classification (Iida et al. 2014) after exercise and drug therapy. When an occlusive lesion was found in the FP vessels on angiography, vascular surgeons and interventional cardiologists discussed EVT indications.

This study protocol was developed in accordance with the Declaration of Helsinki and was approved by the ethics committee.

### Interventions

The ipsilateral or crossover approach was used for EVT. In this approach, 6-French (Fr) or 7-Fr sheaths were inserted, and unfractionated heparin (5000 U) was injected intraarterially. After a 0.014-inch guidewire was passed through the target lesion, intravascular ultrasonography (IVUS) images were recorded by a manual pullback. We measured the distal external elastic membrane (EEM) as the reference vessel diameter, and the Pre-dilatation was sized at 1: 1 with the reference diameter.

A chocolate PTA balloon was expanded to half nominal for 30 s, followed by slow inflation to nominal for an additional 90 s, as described previously (Mustapha et al. 2018). Because this was a retrospective study and there were no reports of appropriate conventional balloon expansion methods, conventional balloons that expand slowly till they achieve the target pressure for approximately 2 min were also used. Conventional balloons were used before the introduction of chocolate PTA balloons for pre-expansion. If there was type D or more severe dissection or residual stenosis greater than 50%, bailout stent placement was performed. If this criterion was not met, DCB was selected as the final device.

### Medical therapy

Dual antiplatelet therapy with aspirin (100 mg/day) plus ticlopidine (200 mg/day), clopidogrel (75 mg/day), or cilostazol (200 mg/day) was prescribed for at least three days before EVT. It was continued for at least one month after that.

### Study definitions and clinical follow-up

Rate of severe dissections was the primary endpoint. Secondary endpoints included percent diameter stenosis (%DS) after pre-dilatation, rate of bailout stenting, primary patency at six months, and freedom from TLR at six months. A clinical evaluation was performed at one, three, and six months. Duplex ultrasonography assessment was performed routinely at one, three, and six months post-EVT to evaluate vessel patency.

When duplex ultrasonography suggested stenosis or occlusion, angiography was performed for confirmation. Restenosis was defined as a peak systolic velocity ratio of > 2.4 on a duplex ultrasound image, which indicated > 50% narrowing, or > 50% diameter stenosis or occlusion on angiography (Ranke et al. 1992). CLTI was defined as described previously (Takahara et al. 2015). All lesions within the FP segment were characterized according to the Trans-Atlantic Inter-Society Consensus-II (TASC-II) classification (Norgren et al. 2007). The proposed Peripheral Arterial Calcium Scoring System (PACSS) was used to categorize the degree of lesion calcification (Rocha-Singh et al. 2014). Below-the-knee (BTK) runoff was assessed using pre-angiography. Poor tibial vessel runoff was defined as runoff in one or no BTK vessel (Soga et al. 2011). All angiograms were evaluated independently by two experienced operators. If there was a disagreement, the more severe pattern was used in the analyses. Angiographic dissection was classified according to the National Heart, Lung and Blood Institute (NHLBI) dissection classification system (Rogers and Lasala 2004), which defines flow-limiting dissection as types E and F. Severe dissection was defined as type C or higher in the NHLBI classification (Fujihara et al. 2017). TLR was defined as a repeated endovascular or surgical procedure for the treated lesion.

### Statistical analysis

Statistical analyses were performed using JMP software version 13.0.0 (SAS Institute, Cary, NC, USA). Continuous variables are reported as mean ± standard deviation and were compared using the *t*-test. Categorical variables were reported as count (percentage) and were compared using chi-squared test or Fisher’s exact test. All baseline values and procedure characteristics were assessed using univariate logistic regression analysis to identify independent predictors of the bailout stent. The consistency of image evaluation was evaluated using the weighted κ statistic. Estimates of primary patency and TLR rates were calculated using the Kaplan–Meier method. Intergroup differences were assessed using the log-rank test. In all the analyses, statistical significance was set at *P* < 0.05.

## Results

### Patients and procedural characteristics

Baseline characteristics are shown in Table 1. There were no significant differences in patient and lesion characteristics. Procedural characteristics are shown in Table 2. In the pre-dilatation balloon, although there was no significant difference in balloon diameters, the balloon length was 90 ± 37 and 148 ± 95 mm (*P* = 0.004). Because a conventional balloon contains a compliance balloon with an average expansion pressure of 8.3 atm and a non-compliance balloon 19.0 atm, the inflation pressure was 11 ± 3 atm and 16 ± 7 atm (*P* < 0.001) in the chocolate PTA and conventional groups, respectively.

**Table 1:** Patient and lesion characteristics

	**Chocolate** ***n* = 48**	**Conventional** ***n* = 63**	*P*
**Patient characteristics**			
Age, years	74 ± 8	74 ± 9	0.900
Male, n (%)	36 (75)	46 (73)	0.814
Body mass index, kg/m^2^	23.4 ± 4.2	23.2 ± 4.0	0.682
Hypertension, n (%)	43 (90)	55 (87)	0.711
Dyslipidemia, n (%)	28 (58)	32 (51)	0.430
Diabetes mellitus, n (%)	31 (65)	41 (65)	0.957
Chronic kidney disease, n (%)	31(65)	46(73)	0.340
Hemodialysis, n (%)	16 (33)	27 (43)	0.308
Smoker, n (%)	10 (21)	12 (19)	0.815
Aspirin, n (%)	34 (71)	48 (76)	0.525
Thienopyridine, n (%)	41 (85)	54 (86)	0.965
Cilostazol, n (%)	12 (25)	10 (16)	0.232
Prior Coronary artery disease, n (%)	16 (33)	28 (44)	0.236
Prior peripheral artery disease, n (%)	22 (46)	31 (49)	0.725
Prior cerebrovascular disease, n (%)	9 (19)	8 (13)	0.380
Chronic limb-threatening ischemia, n (%)	10 (21)	19 (30)	0.268
**Lesion characteristics**			
Pre-ankle brachial index	0.70 ± 0.16	0.65 ± 0.17	0.094
Post-ankle brachial index	0.93 ± 0.15	0.90 ± 0.19	0.744
TASCII C/D, n (%)	20 (42)	33 (52)	0.263
PACSS 3/4, n (%)	24 (50)	33 (52)	0.804
include P2/3 lesion, n (%)	14 (29)	24 (38)	0.326
CTO lesion, n (%)	11 (23)	22 (35)	0.170
Lesion length, mm	134 ± 98	156 ± 96	0.116
Vessel diameter, mm	4.7 ± 0.7	4.7 ± 0.9	0.468
poor run off vessel, n (%)	14 (29)	24 (38)	0.326

*TASCII* Trans-Atlantic Inter-Society Consensus II, *PACSS* Peripheral Arterial Calcium Scoring System, *CTO* Chronic total occlusion

**Table 2:** Procedural and angiographical characteristics

	**Chocolate** ***n* = 48**	**Conventional** ***n* = 63**	*P*
**Procedural characteristics**			
**Pre-balloon dilatation**			
Balloon diameter, mm	4.8 ± 0.8	4.6 ± 0.8	0.156
Balloon length, mm	90 ± 37	148 ± 95	0.004
Inflation pressure, atm	11 ± 3	16 ± 7	< 0.001
Inflation time, sec	154 ± 42	132 ± 77	0.225
**Final device**			
Drug-coated balloon, n (%)	47 (98)	53 (84)	0.016
Bailout stent, n (%)	1 (2.1)	10 (15.9)	0.016
**Angiographical results**			
**Post pre-dilatation**			
Dissection grade			
Grade none, n (%)	29 (60)	18 (29)	0.006
Grade A, n (%)	14 (29)	19 (30)	
Grade B, n (%)	3 (6)	10 (16)	
Grade C, n (%)	1 (2)	8 (13)	
Grade D, n (%)	1 (2)	7 (11)	
Grade E or F, n (%)	0	1(2)	
Severe dissection, n (%)	2 (4.2)	16 (25.4)	0.003
%Diameter stenosis, %	18 ± 15	20 ± 17	0.409
**Final angiogram**			
Severe dissection, n (%)	1 (2.1)	7 (11.1)	0.070
%diameter stenosis, %	13 ± 11	13 ± 12	0.990

### Angiographic outcome after pre-dilatation and the rate of bailout stent

**Fig. 2:**
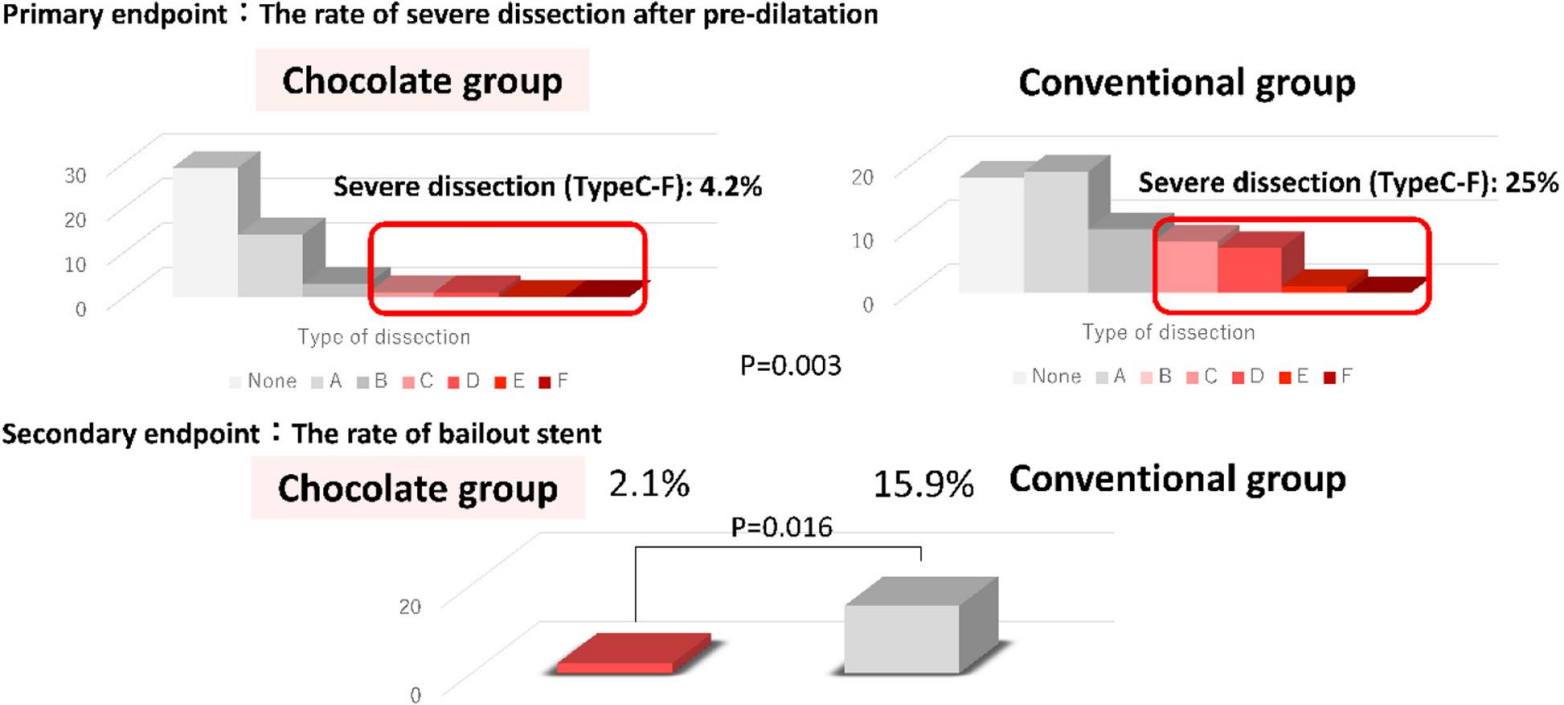
Primary and secondary endpoints

In the angiogram after pre-dilatation, severe dissection was observed in 2 (4.2%) and 16 (25%) lesions (*P* = 0.003), the %DS was 18 ± 15 and 20 ± 17 (*P* = 0.409) (Table 2 and Fig. 2), and the bail-out stent rate was 2.1% (1 lesion) and 15.9% (10 lesions) in the Chocolate PTA and conventional groups, respectively (Table 2 and Fig. 2). The reproducibility of image classification was moderate-high consistent (κ = 0.62). In the univariate analysis, predictors of a bailout stent were chronic total occlusion (CTO) lesions, lesion length, and non-use of Chocolate PTA balloon (Table 4).

### Clinical outcomes

**Fig. 3:**
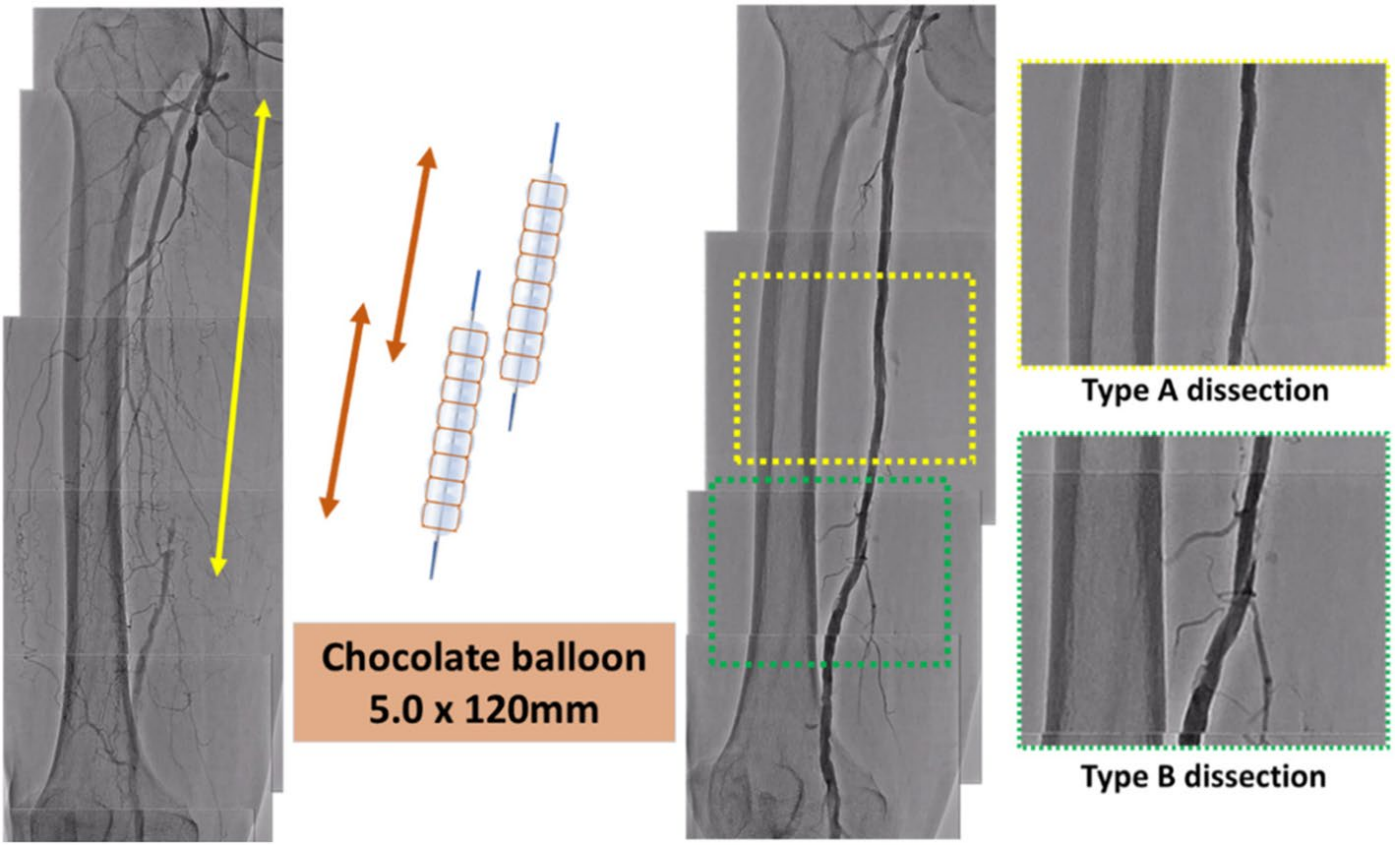
Kaplan–Meier analysis of primary patency and TLR at six months

In the Chocolate PTA group, rates of primary patency and freedom from TLR at six months were 93.1% and 83.9%, respectively; in the conventional group, the rates were 97.6% and 89.2%, respectively. There were no significant differences between the two groups (Fig. 3).

## Discussion

**Fig. 4:**
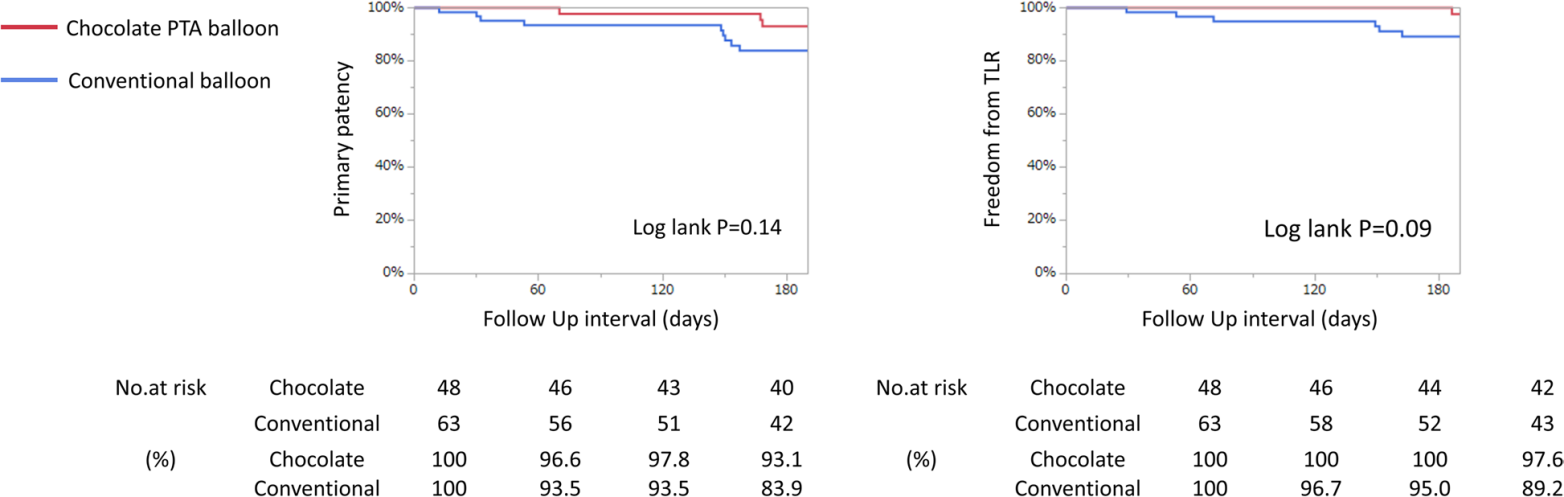
Representative case. In the SFA long CTO case, vessel preparation was successful in the Chocolate balloon and could be treated with DCB. SFA, superficial femoral artery; CTO, chronic total occlusion; DCB, drug-coated balloon

This study is the first to evaluate the impact of Chocolate PTA balloon for vessel preparation with the DCB strategy compared to other conventional balloons. This study found that the Chocolate PTA balloon had less vessel dissection and sufficient expansion than the conventional balloon. A representative case is illustrated in Fig. 4.

In recent years, the development of DCB has resulted in favorable outcomes and has contributed greatly to revascularization without stent implantation. Tepe et al. (Tepe et al. 2015) demonstrated that DCB was associated with a higher primary patency at 12 months than conventional balloon angioplasty in a randomized controlled trial (82.2% vs. 52.4%, *P* < 0.01). However, 7%–12% of patients require provisional stent implantation; therefore, the DCB strategy is essential to obtain a sufficient lumen area using uncoated balloon dilatation and to prevent flow-limiting dissection. To achieve successful EVT using the DCB strategy, balloon angioplasty must be optimized.

The frequency of severe angiographic dissection after pre-dilatation was 4.2% in the Chocolate PTA group and 25% in the conventional group (Fig. 2). In a previous study (Mustapha et al. 2018), the incidence of type C or higher dissection was 11.1%. Their findings were comparable to ours, although we could not compare them directly. IVUS findings showed a similar minimum lumen area in both groups after pre-dilatation, but the incidence of dissection above 180° was lower in the Chocolate PTA balloon group (Table 3).

**Table 3:** IVUS parameters

**IVUS parameters**	**Chocolate** ***n* = 48**	**Conventional** ***n* = 63**	*P*
**Distal reference**			
Reference diameter, mm	5.1 ± 0.8	5.1 ± 0.8	0.621
Distal EEM CSA, mm^2^	20 ± 7	21 ± 7	0.394
Distal lumen CSA mm^2^	15 ± 7	15 ± 6	0.916
**Post pre-dilatation**			
Dissection ≥ 180°, n (%)	2 (6)	11 (22)	0.014
Minimum lumen area, mm^2^	11.4 ± 3.7	12.1 ± 4.9	0.701

*EEM* External elastic membrane, *CSA* Cross sectional area, *IVUS* Intravascular ultrasound

A previous study (Fujihara et al. 2017) reported an incidence of severe dissection of 42%, with reference vessel diameter < 5 mm, lesion length > 15 cm, and CTO lesion as predictors. We observed no significant differences in the lesion characteristics of reference diameter, lesion length, and the ratio of CTO (Table 1). The effect of the Chocolate PTA balloon might have prevented the severe dissection.

There are several reasons for this effect. First, Chocolate PTA balloon is a novel, semi-compliant balloon catheter aimed at less traumatic percutaneous transluminal angioplasty (Spiliopoulos et al. 2019; Ward and Mena-Hurtado 2014). The Chocolate PTA balloon is a balloon constrained by a mounted nitinol structure surrounding it. This nitinol cage prevents vessel injury from torsional stress that causes dissections. It also prevents partial overdilation of the balloon, distributes the pressure on the vessel, and minimizes arterial wall stress on vessel.

Second, a previous study (Tan et al. 2018) demonstrated that long balloons covering CTO lesions have less dissection, suggesting the possibility of dissection at the balloon edge. We observed a shorter balloon length in the Chocolate PTA balloon group. However, fewer dissections and the Chocolate PTA balloon nitinol cage prevent longitudinal balloon elongation, which may reduce edge dissection.

Third, regarding preventing vascular dissection in balloon dilation, a previous study (Sugihara et al. 2020) performed super slow inflation that dilated the balloon slowly at a low pressure. In this study, the Chocolate PTA balloon uses the method of expansion in the Chocolate Bar registry (Mustapha et al. 2018), which "extends to half nominal in 30 s and then increases to nominal pressure." Conversely, the conventional group raises the nominal pressure by approximately 10 s according to the decision of each operator. Although different from the protocol of super slow inflation (Sugihara et al. 2020), slower balloon inflation in the Chocolate PTA group than in the conventional group may be one of the factors that causes less severe dissection.

Another finding of our study is that the use of Chocolate PTA balloon may prevent bailout stents. The rate of bailout stenting in the Chocolate PTA balloon group was 2.1%, which was a low probability in the Chocolate PTA balloon group, considering that it was 15.9% in the conventional group (Fig. 2).

Predictors of bailout stents were CTO lesion, longer lesion length, and non-use of Chocolate PTA balloon (Table 4). In a previous study (Sirignano et al. 2018), using a Chocolate PTA balloon for vessel preparation before DCB, many complex lesions contained 65.5% CTO lesions and 21.4% of CTOs with a lesion length > 150 mm. However, the bailout stent rate was 9.5%. Randomized controlled trials comparing bare nitinol stents with plain old balloon angioplasty (POBA) reported a bailout stent frequency of up to 40% in the PTA group (Schillinger et al. 2006; Laird et al. 2010). In addition, randomized controlled trials comparing DCB and POBA reported that the bailout stent rate was 7.3%–26.7% (Tepe et al. 2015; Zeller et al. 2015). Although direct comparisons could not be made, the Chocolate PTA balloon group in this study, even in treating complex lesions (TASC-II C/D 42%, CTO 23%, PACSS 3/4 50%, and lesion length 134 ± 98 mm), had a low rate of bailout stenting.

**Table 4:** Logistic regression analysis of bailout stent

Variables	Univariate analysis		
	OR	95% CI	*P* value
Chronic total occlusion	**10.8**	**2.8–42.1**	0.001
Lesion length (per 10-mm increase)	**1.08**	**1.02–1.15**	0.045
Non-use of Chocolate PTA balloon (vs Conventional)	**8.9**	**1.1–71.9**	0.009

*OR* Odds ratio, *CI* Confidence interval

This table shows the items with a statistical difference in the univariate analysis

### Relationship between vessel preparation by Chocolate PTA balloon and clinical outcomes

After six months, there were no significant differences in primary patency and freedom from TLR between the two groups (Fig. 4). This may be because if a severe dissection was achieved by pre-dilation, bail-out stenting did not make a significant difference between residual stenosis and severe incision on final angiography.

### Limitations

This study has several limitations. First, it was a small, retrospective, single-center study. Second, grouping according to the period may have included potential bias. Third, since there were few cases of bailout stents, the factor can only be investigated by univariate analysis. Thus, it is necessary to increase the number of events and perform a multivariate analysis. There is a need to increase this number. Finally, the observation period was only six months, and the long-term prognosis is unknown. Further investigation is required to clarify the long-term prognosis.

## Conclusion

Chocolate PTA balloons can reduce the rate of severe dissection while maintaining a sufficient dilatation effect in DCB vessel preparation compared to conventional balloons.

## Data Availability

All data are referenced from the medical records and stored in the hospital. It can be published if necessary.

## Abbreviations

EVT: Endovascular treatment
FP: Femoropopliteal
DCB: Drug-coated balloon
SFA: Superficial femoral artery
PTA: Percutaneous transluminal angioplasty
TLR: Target-lesion-revascularization
CLTI: Chronic limb-threatening ischemia
Fr: French
IVUS: Intravascular ultrasonography
%DS: Percent diameter stenosis
TASC-II: Trans-Atlantic Inter-Society Consensus-II
NHLDI: The National Heart, Lung and Blood Institute
CTO: Chronic total occlusion
POBA: Plain old balloon angioplasty
